# A New Measurement of Internet Addiction Using Diagnostic Classification Models

**DOI:** 10.3389/fpsyg.2017.01768

**Published:** 2017-10-10

**Authors:** Dongbo Tu, Xuliang Gao, Daxun Wang, Yan Cai

**Affiliations:** School of Psychology, Jiangxi Normal University, Nanchang, China

**Keywords:** measurement, diagnostic classification models, internet addiction, symptom criteria-level information, cognitive diagnosis models

## Abstract

To obtain accurate, valid, and rich information from the questionnaires for internet addiction, a diagnostic classification test for internet addiction (the DCT-IA) was developed using diagnostic classification models (DCMs), a cutting-edge psychometric theory, based on DSM-5. A calibration sample and a validation sample were recruited in this study to calibrate the item parameters of the DCT-IA and to examine the sensitivity and specificity. The DCT-IA had high reliability and validity based on both CTT and DCMs, and had a sensitivity of 0.935 and a specificity of 0.817 with AUC = 0.919. More important, different from traditional questionnaires, the DCT-IA can simultaneously provide general-level diagnostic information and the detailed symptom criteria-level information about the posterior probability of satisfying each symptom criterion in DMS-5 for each patient, which gives insight into tailoring individual-specific treatments for internet addiction.

## Introduction

Diagnostic classification models (DCMs; Rupp et al., 2010), also commonly called cognitive diagnosis models (CDMs), provide an alternative psychometric framework that can be used for test development, psychometric analysis, and detailed score reporting. DCMs are special cases of latent class models that characterize the relationship of observable response data to a set of categorical latent variables, which might be more suitable than classical test theory (CTT) and item response theory (IRT) models when latent constructs are multidimensional and finer-grained. As mentioned by de la Torre et al. (2015), the CDMs make it possible to optimally use information in diagnosis and can obtain the interactions among attributes while IRT cannot. With this approach (CDMs) we can investigate to which extent a symptom—as described by an item will be observed given the various combinations of multiple disorders (de la Torre et al., 2015). More important, different from the IRT models, DCMs have the potential to provide diagnostic reports at the symptom level by linking the instruments with some well-established diagnostic system, such as, the 5th edition of the Diagnostic and Statistical Manual of Mental Disorders (DSM-5; American Psychiatric Association, 2013).

Although most of the developments and applications of DCMs have taken place in Education with the intention of identifying students' skill profiles, these models have sufficient generality to be applied to psychological disorder diagnosis to identify individuals' disorder profiles or symptom profiles (e.g., Jaeger et al., 2006; Templin and Henson, 2006; de la Torre et al., 2015).

Internet addiction (IA) is a frequent psychology disorder in DSM-5. Although numerous IA instruments have been developed internationally to assess IA, there are still large rooms to improve for the diagnosis and assessment of IA. On one hand, a large number of instruments of IA, such as, the Young's (1998a,b) Internet Addiction Test (IAT), classify individuals as IA mainly based on the total score or transformed total score, which cannot provide more detailed information of each individual's symptom spectrum of IA and cannot give good insight into tailoring individual-specific treatments for IA. On the other hand, many instruments are not developed based on DSM-5. A close scrutiny of the IAT reveals that not all criteria in the DSM-5 are measured, and the same can be said of many other instruments for IA. Finally, although some researchers (such as Watters et al., 2013) have considered the multidimensional structure of IA, the internal structures are typically not directly related to the symptom criteria defined in the DSM-5. Therefore, the instruments cannot provide symptom level reports.

To achieve the above aims, in this study, a diagnostic classification test for internet addiction (DCT-IA) is developed under the DCMs framework based on DSM-5 to obtain both general and detailed information about diagnosis, symptom spectrum, and treatment of internet addition. Currently the DSM-5 has published the diagnosis standard of IA, which has been widely accepted and used to guide diagnosis and treatment of IA in practical applications, therefore we used the DSM-5 to guide the development of CDT-IA. A series of psychometric analyses has been conducted as well to demonstrate the current and potential value of DCMs in psychological assessment. It is worth emphasizing that one major benefit of using DCMs is that we can estimate each individual's symptom profile and the posterior probability of internet addiction (PPIA) according to the DSM-5. This information could be very valuable for diagnostic and interventional purposes.

## Methods

### Diagnostic criteria for internet addiction

Currently, the well-known systems of diagnostic criteria for internet gaming disorder (also commonly referred to as IA, internet use disorder, or gaming addiction) is defined in the DSM-5. There are nine symptom criteria shown in Table 1, those who meet five or more symptom criteria are defined as internet gaming disorder by DSM-5.

**Table 1:** Symptom criteria of internet gaming disorder defined in DSM-5.

**ID**	**Symptom criteria**
S1	Preoccupation with Internet (games)
S2	Withdrawal symptoms when Internet (gaming) is taken away
S3	Tolerance—the need to spend increasing amounts of time engaged in Internet (games)
S4	Unsuccessful attempts to control the participation in Internet (games)
S5	Loss of interests in previous hobbies and entertainment as a result of, and with the exception of, Internet (games)
S6	Continued excessive use of Internet (games) despite knowledge of psychosocial problems
S7	Has deceived family members, therapists, or others regarding the amount of Internet gaming
S8	Use of Internet (games) to escape or relieve a negative mood
S9	Has jeopardized or lost a significant relationship, job, or educational or career opportunity because of participation in Internet (games)

Given that (1) the symptom criteria of IA defined in DSM-5 were widely accepted and used to guide diagnosis in practice, and (2) borrowing the idea or approach from Templin and Henson's (2006) diagnosis of psychological disorder via DCM, these symptom criteria of IA in DSM-5 were used as the latent attributes/variables in DCMs to make diagnosis in this study. These symptom criteria were person parameters need to be estimated to reflect his/her symptom profile and calculate the PPIA, which is the probability of processing five or more symptom criteria of IA defined in DSM-5, for each person. In this study, IA was a general concept according to Young's (1999) research, mainly referring as to internet use disorder but not only limited to internet gaming disorder. However, the nine symptom criteria in DSM-5 were borrowed here to define the IA or internet use disorder.

### Diagnostic classification models (DCMs)

Under the DCMs framework, symptom criteria are typically treated as latent variables with two statuses—presence or absence. *K* symptom criteria in an instrument will yield 2^*K*^ symptom profiles, and each symptom profile is related with one unique latent class. The symptom profile for latent class *c* is denoted as α_*c*_ = (α_*c*1_,⋯, α_*ck*_,⋯, α_*cK*_), where α_*ck*_ = 1 if individuals in latent class *c* have met symptom criteria *k* and 0 if not. DCMs aim to build connections between individuals' item responses and their symptom profiles.

A great quantity of DCMs were developed in the literature and they differ in various ways, such as, how to model the interaction among symptom criteria. Some models are referred to as saturated models because they consider all possible interactions among symptom criteria, such as, the general diagnostic model (GDM; von Davier, 2008), log-linear (LDCM; Henson et al., 2009), and generalized deterministic input, noisy, “and” gate (G-DINA; de la Torre, 2011) model. Some models assume that symptom criteria interact in some particular manners and thus referred to as special or reduced models. For item *j*, only the measured symptom criteria have an impact on the endorsement probability. The profile of symptom criteria measured by item *j* is denoted as $\alpha_{lj}^{*}=(\alpha_{l1,}\cdots ,\alpha_{lk,}\cdots ,\alpha_{lK_{j}^{*}})$, where $K_{j}^{*}$ is the number of criteria measured by item *j*. The item response function of the G-DINA mode (de la Torre, 2011) is expressed as:

(1) $$\begin{array}{rcl} P\left(X_{j}=1|\alpha_{lj}^{*}\right) & = & \phi_{j0}+\overset{K_{j}^{*}}{\underset{k=1}{\sum }}\phi_{jk}\alpha_{lk}+\overset{K_{j}^{*}}{\underset{k\prime =k+1}{\sum }}\overset{K_{j}^{*}-1}{\underset{k=1}{\sum }}\\ \phi_{jkk\prime }\alpha_{lk}\alpha_{lk\prime }+\cdots +\phi_{j12\ldots K_{j}^{*}}\overset{K_{j}^{*}}{\underset{k=1}{\prod }}\alpha_{lk}, \end{array}$$

where $P\left(X_{j}=1|\alpha_{lj}^{*}\right)$ is the endorsement probability of examinees with the latent symptom profile $\alpha_{lj}^{*}$, *ϕ*_*j*0_ is the intercept for item *j*, *ϕ*_*jk*_ is the main effect due to having symptom *k*, $\phi_{jkk\prime }$ is the interaction effect due to having symptoms *k* and *k*′, $\phi_{j12\ldots K_{j}^{*}}$ is the interaction effect due to having symptoms 1 up to $K_{j}^{*}$.

The addictive cognitive diagnosis model (ACDM; de la Torre, 2011) is a special case of G-DINA model by supposing no interaction effects. ACDM can be formulated as

(2) $$\begin{array}{r} P\left(X_{j}=1|\alpha_{lj}^{*}\right)=\phi_{j0}+\overset{K_{j}^{*}}{\underset{k=1}{\sum }}\phi_{jk}\alpha_{lk}. \end{array}$$

The linear logistic model (LLM; Maris, 1999), the logit-link G-DINA model without interaction terms, is expressed as

(3) $$\begin{array}{rcl} \log it\left[P\left(X_{j}=1|\alpha_{lj}^{*}\right)\right] & = & \phi_{j0}+\overset{K_{j}^{*}}{\underset{k\;=\;1}{\sum }}\phi_{jk}\alpha_{lk},or,P\left(X_{j}=1|\alpha_{lj}^{*}\right)=\\ \frac{\exp\left(\phi_{j0}+\overset{K_{j}^{*}}{\underset{k\;=\;1}{\sum }}\phi_{jk}\alpha_{lk}\right)}{1+\exp\left(\phi_{j0}+\overset{K_{j}^{*}}{\underset{k\;=\;1}{\sum }}\phi_{jk}\alpha_{lk}\right)}. \end{array}$$

The reduced reparametrized unified model (RRUM; Hartz et al., 2002) is the log-link G-DINA model without interaction terms and it is formulated as

(4) $$\begin{array}{r} \log\left[P\left(X_{j}=1|\alpha_{lj}^{*}\right)\right]=\phi_{j0}+\overset{K_{j}^{*}}{\underset{k\;=\;1}{\sum }}\phi_{jk}\alpha_{lk}. \end{array}$$

The DINA (deterministic inputs, noisy, “and” gate; Junker and Sijtsma, 2001) is also a special case of G-DINA model by setting all the parameters, except ϕ_*j*0_ and $\phi_{j12\ldots K_{j}^{*}}$, to zero, as following:

(5) $$\begin{array}{r} P\left(X_{j}=1|\alpha_{lj}^{*}\right)=\phi_{j0}+\phi_{j12\ldots K_{j}^{*}}\overset{K_{j}^{*}}{\underset{k\;=\;1}{\prod }}\alpha_{lk}. \end{array}$$

By setting $\phi_{jk}=-\phi_{jkk^{\prime }}=\cdots ={\left(-1\right)}^{K_{j}^{*}+1}\phi_{j12\ldots K_{j}^{*}}$, and the DINO (deterministic input, noisy, “or” gate; Templin and Henson, 2006) model is obtained from the G-DINA model as

(6) $$\begin{array}{r} P\left(X_{j}=1|\alpha_{lj}^{*}\right)=\phi_{j0}+\phi_{jk}\alpha_{lk}. \end{array}$$

The G-DINA model and the five reduced model were employed in this article in that these models are very typical and most representative models of DCMs and they are relative widely used in psychological disorder assessment (e.g., Jaeger et al., 2006; Templin and Henson, 2006; de la Torre et al., 2015). The parameters of the G-DINA model and all reduced models can be estimated using the marginal maximum likelihood estimation (MMLE) algorithm.

### Diagnostic classification test for internet addiction (DCT-IA)

Given that most existing self-reported IA questionnaires can neither measure all the IA symptom criteria defined in the DSM-5, nor provide the information at the level of the IA symptom criteria, this study aims to develop a DCT-IA to measure the IA symptom criteria in the DSM-5 and to diagnose IA and diagnose the presence or absence of each symptom criteria.

The DCT-IA originally consisted of 181 items carefully selected based on the IA symptom criteria in the DSM-5 from ten self-rating inventories, including the IAT (Young, 1998a,b), Internet Related Problem Scale (IRPS; Armstrong et al., 2000), Pathological Use Scale (PIU; Morahan-Martin and Schumacher, 2000), Online Cognition Scale (OCS; Davis et al., 2002), Internet Addiction Test (IAT; Widyanto and McMurran, 2004), short version of IAT (s-IAT; Pawlikowski et al., 2013); also included four inventories developed by Chinese researchers, which are Adolescent Pathological Internet Use Scale (APIUS; Lei and Yang, 2007), Chinese Internet Addiction Scale (CIAS; Chen et al., 2003), Internet Addiction Scale (IAS; Yang and Zheng, 2008), and Computer Game Addiction Scale (CGAS; Liu and Li, 2007), respectively. These 181 selected items measure all nine IA symptom criteria in DSM-5. Items were modified to refer to the previous 12-month and to have consistent two response categories—yes or no. Each item measures at least one symptom. An item by criterion association matrix or Q-matrix (Tatsuoka, 1990), as shown in Table 2, was constructed by twelve experts, who were divided into four groups with each group constructing about 46-item-Q-matrix. Each group includes two psychotherapists with more than 3 years of clinical experience on IA and one expert with 3-year research experience in the measurement of IA.

**Table 2:** Q-matrix for the part items of DCT-IA.

**Item**	**Symptom criterion of depression**								
	**S1**	**S2**	**S3**	**S4**	**S5**	**S6**	**S7**	**S8**	**S9**
1	1	0	0	0	0	1	0	0	1
2	0	0	0	0	0	1	0	0	1
3	0	0	1	0	0	0	0	0	0
4	0	0	0	1	0	0	0	0	0
5	1	0	0	0	1	0	0	0	0
6	1	0	0	0	0	1	0	0	1
7	1	0	0	0	0	1	0	0	0
8	0	0	0	0	1	0	0	0	0
9	0	1	0	0	0	0	0	0	0
10	0	0	0	0	0	0	0	0	1
11	0	0	1	0	0	1	0	0	0
12	0	0	0	1	0	0	0	0	0
13	0	0	0	0	0	1	0	0	1
14	0	1	0	0	0	0	0	0	0
15	0	0	0	0	0	0	0	0	1

*Criterion S1 to S9 represents the nine symptom criteria for internet addiction defined in the DSM-5. Element 1 in row j and column k in the Q-matrix indicates that item j measures symptom k, whereas element 0 indicates that item j does not measure symptom k*.

In the Q-matrix, entry 1 indicates a symptom criterion is measured by the item and entry 0 indicates not. The construction of Q-matrix was based on a Delphi method, including three steps. In Step 1, each expert defined the Q-matrix individually. In Step 2, the experts were anonymously provided with the decisions of the other experts in the same group and were told they could change their initial specifications. In Step 3, the three experts in each group met in person, and they discussed in detailed their opinions to form the consistent opinion. However, experts had diverse opinions toward the construction of 27 items. After deleting the 27 items, a 154 item-by-symptom Q-matrix was constructed, and per item measured an average of 1.46 symptom criteria and per symptom criteria was measured by an average of 14.1 items.

Table 3 gives some item examples in the DCT-IA. Item “Use internet to avoid or alleviate helplessness, guilty or anxiety” measures “Use of Internet to escape or relieve a negative mood” (S8). While item “Even though there are times when I would like to, I can't cut down on” measures “Tolerance—the need to spend increasing amounts of time engaged in Internet” (S3) and “Unsuccessful attempts to control the participation in Internet” (S4).

**Table 3:** Some item examples in DCT-IA.

**Items (or abbreviated content)**	**Q-matrix**								
	**S1**	**S2**	**S3**	**S4**	**S5**	**S6**	**S7**	**S8**	**S9**
Use internet to avoid or alleviate helplessness, guilty or anxiety	0	0	0	0	0	0	0	1	0
Even though there are times when I would like to, I can't cut down on my use of the internet	0	0	1	1	0	0	0	0	0
Use internet more than ought to	0	0	1	0	0	1	0	0	0
Use of internet affecting learning	0	0	0	0	0	0	0	0	1
Ignore what should do	1	0	0	0	0	1	0	0	0

*Criterion S1 to S9 represents the nine symptom criteria for internet addiction defined in the DSM-5. Element 1 in row j and column k in the Q-matrix indicates that item j measures symptom k, whereas element 0 indicates that item j does not measure symptom k*.

### Participant sample

A total of 1,558 Participants with and without IA were recruited for this study. These participants' age ranges from 12 to 36 with mean = 16.2 (*SD* = 4.56), the male-to-female ratio was 43.6:56.4%. Responses of 1,263 individuals were used to calibrate the item parameters of the DCT-IA via DCMs. The rest 295 individuals were recruited as a validation sample to examine the sensitivity and specificity of DCT-IA. The validation sample had two groups, including healthy control group (*N*_1_ = 199) and IA group (*N*_2_ = 96).

The IA group was recruited according to the following exclusion criteria: (1) history of IA, attention-deficit/hyperactivity disorder (ADHD), or obsessive-compulsive disorder (OCD) over the past year; (2) except for use of internet for required activities in a business or profession, averagely spending <3 h on internet each day; and (3) use of internet for pathological gambling. Furthermore, the IA group satisfies that: (1) they all reported surfing internet severely disrupted their normal activities; (2) they all reported that they averagely spent more than 5 h on internet except for required activities in a business or profession each day; and (3) they all defined as IA by IAT (Young, 1998b).

The study also had exclusion criteria to screen the healthy group: (1) averagely spending more than 2 h on internet except for required activities in a business or profession each day; and (2) any diagnosis or treatment for psychiatric illness over the past 24 months. This study was carried out in accordance with the recommendations of ethics committee. All participants gave their written informed consent. The parental consent was also obtained for all participants under the age of 16.

### Statistical analysis

The statistical analysis mainly included four steps, which were explained more details as following.

Step 1: Select the most appropriate DCM for the DCT-IA via Wald test based on the real data.

Selecting appropriate model is considered one of the most important procedures to make valid inferences. A large number of DCMs have been developed, but it is not always clear which model should be used for a given data set. de la Torre (2011) proposed to evaluate whether the reduced model can be used in place of the saturated model without significant loss in model data fit via the Wald test, and Ma et al. (2016) showed that the selected models via the Wald test performed better than, or at least as well as, the saturated model in terms of person parameter estimation. Five reduced models (i.e., rRUM, DINA, DINO, *ACDM*, and LLM), in this paper, were considered. The reduced DCM with a significant *p*-value is deemed acceptable for an item. If more than one reduced DCM is acceptable, the model with the largest *p*-value was chosen as the most appropriate one.

Step 2: Analyze the psychometric characteristics of each item in DCT-IA employing the selected DCM in the step 1.

After selecting the most appropriate model for each item, psychometric characteristics (i.e., item-fit, differential item functioning-DIF, and discrimination), were analyzed for each item. The *S*−*X*_2_ item fit statistic (Orlando and Thissen, 2000, 2003) was used to exam item fit and the Wald test statistic (Hou et al., 2014) was used to detect DIF in different groups (e.g., female and male; rural and urban); then the discrimination index suggested by de la Torre (2008) was calculated (see Formula 7).

Step 3: Choose high-quality items to develop the final DCT-IA based on the statistical indexes including discrimination, model-fit, differential item functioning (DIF) and so on in step 2.

Item selection mainly was conducted based on the statistical indexes including discrimination, model-fit and DIF in step 2. Finally, low discriminating items (<0.45), DIF items and items with poor item fit (*p* < 0.05) were excluded. This procedure was repeated until no item was excluded. GDINA R package (Ma and de la Torre, 2016) was used for model estimation, model selection, and DIF detection. Custom-written code in R (R Core Team, 2016) was used for all other analyses.

(7) $$\begin{array}{r} Disc_{j}=P\left(X_{j}=1|\alpha_{lj}^{*}=1\right)-P\left(X_{j}=1|\alpha_{lj}^{*}=0\right), \end{array}$$

where $P\left(X_{j}=1|\alpha_{lj}^{*}=1\right)$ is the endorsement probability for respondents who have all the symptom criteria measured by item *j*, and $P\left(X_{j}=1|\alpha_{lj}^{*}=0\right)$ is the endorsement probability for respondents who have none of the symptom criteria measured by item *j*.

Step 4: Evaluate the reliability and validity of the final DCT-IA.

As to reliability, the coefficients of Cronbach's alpha and Guttman Split-Half based on CTT, and the symptom-level classification consistency reliability indices (Cui et al., 2012) based on DCMs were both calculated for DCT-IA. Criterion-related validity and convergent validity were examined. More specifically, criterion-related validity and convergent validity were quantified by the coefficients of correlation between the DCT-IA and the IAT (Young, 1998b) and APIUS (Lei and Yang, 2007). Validity evidence was also collected through the cross-validation using a validation sample.

## Results

### Item analysis of the DCT-IA

Table 4 gives the final selected 50 items for the DCT-IA after 104 items were excluded for statistical reasons (such as, discrimination <0.45, poor model-fit and having DIF). The discrimination of items varies from 0.452 to 0.642 with average of 0.550, which clearly shows the remaining 50 items all have a very high discrimination of item response probability between individuals who possessing and absent symptom criteria measured by item. The DCT-IA measure all nine symptom criteria for IA defined in the DSM-5. The number of items measuring each symptom criteria varies from 5 to 10 with an average of 7.6 and an average of 1.37 symptom criteria is assigned per item

**Table 4:** The 50 selected items of the final DCT-IA.

**Number of items**	**Selected model**	**Discrimination**	**Model-fit (item level)**			**DIF (Female and male)**			**DIF (Rural and urban)**		
			**S-*X*^2^**	***df***	***p***	**Wald stat**.	***df***	***p***	**Wald stat**.	***df***	***p***
1	ACDM	0.597	164.60	137	0.054	0.00	8	1.00	4.88	8	0.77
2	ACDM	0.562	148.81	138	0.25	0.04	4	1.00	1.82	4	0.77
3	GDINA	0.560	167.17	139	0.052	0.00	2	1.00	0.24	2	0.89
4	GDINA	0.578	151.87	139	0.215	0.00	2	1.00	0.04	2	0.98
5	ACDM	0.545	144.11	138	0.344	0.00	4	1.00	2.58	4	0.63
6	ACDM	0.563	173.71	137	0.019	0.00	8	1.00	6.47	8	0.60
7	LLM	0.544	155.55	138	0.146	0.00	4	1.00	4.78	4	0.31
8	GDINA	0.513	164.32	139	0.07	0.02	2	0.99	0.11	2	0.95
9	GDINA	0.463	166.15	139	0.058	0.00	2	1.00	0.12	2	0.94
10	GDINA	0.564	145.50	139	0.336	0.03	2	0.99	0.22	2	0.90
11	ACDM	0.567	155.15	138	0.151	0.00	4	1.00	1.73	4	0.79
12	GDINA	0.516	135.98	139	0.557	0.01	2	0.99	0.07	2	0.97
13	RRUM	0.507	152.78	138	0.184	0.00	4	1.00	1.27	4	0.87
14	GDINA	0.530	166.22	139	0.057	0.00	2	1.00	0.16	2	0.92
15	GDINA	0.517	154.36	139	0.176	0.02	2	0.99	0.87	2	0.65
16	LLM	0.528	162.60	138	0.075	0.00	4	1.00	5.04	4	0.28
17	GDINA	0.598	152.26	139	0.209	0.00	2	1.00	0.16	2	0.92
18	GDINA	0.577	152.82	139	0.2	0.00	2	1.00	0.04	2	0.98
19	GDINA	0.570	134.97	139	0.581	0.00	2	1.00	0.08	2	0.96
20	GDINA	0.560	171.56	139	0.032	0.00	2	1.00	0.02	2	0.99
21	GDINA	0.616	171.87	139	0.03	0.00	2	1.00	0.08	2	0.96
22	RRUM	0.452	174.72	138	0.019	0.00s	4	1.00	0.16	4	1.00
23	GDINA	0.610	140.34	139	0.452	0.01	2	1.00	0.26	2	0.88
24	GDINA	0.457	141.02	139	0.436	0.00	2	1.00	0.14	2	0.93
25	GDINA	0.584	118.76	139	0.892	0.00	2	1.00	0.29	2	0.87
26	GDINA	0.575	146.74	139	0.31	0.01	2	1.00	0.00	2	1.00
27	RRUM	0.594	164.89	138	0.059	0.02	4	1.00	0.61	4	0.96
28	GDINA	0.497	180.71	139	0.01	0.00	2	1.00	1.16	2	0.56
29	GDINA	0.598	137.23	139	0.526	0.01	2	0.99	0.08	2	0.96
30	LLM	0.589	163.66	138	0.067	0.02	4	1.00	2.03	4	0.73
31	GDINA	0.556	125.27	139	0.792	0.00	2	1.00	0.03	2	0.99
32	GDINA	0.568	146.82	139	0.308	0.00	2	1.00	0.42	2	0.81
33	GDINA	0.536	140.87	139	0.44	0.00	2	1.00	1.30	2	0.52
34	GDINA	0.603	162.37	139	0.085	0.01	2	1.00	1.01	2	0.60
35	GDINA	0.580	161.98	139	0.089	0.00	2	1.00	0.76	2	0.69
36	DINO	0.452	161.34	139	0.094	0.00	4	1.00	0.60	4	0.96
37	LLM	0.526	172.28	138	0.025	0.00	4	1.00	0.76	4	0.94
38	GDINA	0.554	104.15	139	0.988	0.00	2	1.00	0.06	2	0.97
39	GDINA	0.459	135.43	139	0.57	0.00	2	1.00	0.52	2	0.77
40	LLM	0.607	143.94	138	0.347	0.00	4	1.00	0.46	4	0.98
41	GDINA	0.542	161.09	139	0.097	0.00	2	1.00	0.96	2	0.62
42	GDINA	0.526	132.47	139	0.64	0.00	2	1.00	0.03	2	0.98
43	LLM	0.525	162.81	138	0.073	0.00	4	1.00	5.92	4	0.21
44	GDINA	0.585	175.30	139	0.02	0.00	2	1.00	0.08	2	0.96
45	GDINA	0.635	179.10	139	0.012	0.00	2	1.00	0.06	2	0.97
46	GDINA	0.642	171.92	139	0.03	0.00	2	1.00	0.03	2	0.98
47	GDINA	0.508	145.39	139	0.338	0.01	2	1.00	0.03	2	0.99
48	GDINA	0.576	161.88	139	0.09	0.01	2	1.00	0.14	2	0.93
49	GDINA	0.505	170.26	139	0.037	0.00	2	1.00	0.20	2	0.90
50	ACDM	0.502	150.20	138	0.226	0.01	4	1.00	0.42	4	0.98

*DIF, differential item functioning*.

Sixteen items measure more than one symptom of IA in DSM-5 while the others all measure only one symptom. One general model (G-DINA model) and four reduced model are finally selected by DCT-IA based on item-level model-fit index (Wald statistics). For four reduced model, six-ACDM, six-LLM, three-RRUM, and one DINO models are selected by sixteen items which measure more than one symptom.

Here, two example items were provided. For the first example item, “Empty, boring and uninteresting without internet” (measuring S1 and S5), with the chosen DINO model, this item response probability was expressed as

(8) $$\begin{array}{r} P\left(X_{j}=1|\alpha_{lj}^{*}\right)=\phi_{j0}+\phi_{jk=1\;or\;5}\alpha_{lk=1\;or\;5}, \end{array}$$

where ϕ_*j*0_ was the baseline probability; ϕ_*jk* = 1 *or* 5_ was the main effects (non-negative) of S1 or S5. This showed those who had either S1 (Preoccupation with Internet) or S5 (Loss of interests in previous hobbies and entertainment as a result of, and with the exception of, Internet) would had a high probability (equal to ϕ_*j*0_ + ϕ_*jk* = 1 *or* 5_) to response “Yes” for “Empty, boring and uninteresting without internet,” while those who had neither S1 and S5 would had a low probability (equal to ϕ_*j*0_) to response “Yes.”

For the second item, “Use internet more than ought to” (measuring S3 and S6), with the chosen ACDM, this item response probability was expressed as

(9) $$\begin{array}{r} P\left(X_{lj}=1|\alpha_{lj}^{*}\right)=\phi_{j0}+\phi_{j3}\alpha_{l3}+\phi_{j6}\alpha_{l6}, \end{array}$$

where ϕ_*j*3_ and ϕ_*j*6_ were the two main effects (non-negative) of S3 and S6, respectively. There were two main effects but no interaction effect between criterion S3 and S6. The item response probabilities (IRP) were ϕ_*j*0_, ϕ_*j*0_ + ϕ_*j*3_α_*l*3_, ϕ_*j*0_ + ϕ_*j*6_α_*l*6_, and ϕ_*j*0_ + ϕ_*j*1_α_*l*1_ + ϕ_*j*6_α_*l*6_ for those who had neither S3 nor S6, those who had S3, those who had S6 and those who had both S3 and S6, respectively. That was to say those who had more criterions of S3 (“Tolerance—the need to spend increasing amounts of time engaged in Internet”) and S6 (“Continued excessive use of internet despite knowledge of psychosocial problems”) would have higher probability to say “Yes” for “Use internet more than ought to.”

### Reliability and validity

The coefficients of Cronbach's alpha and Guttman split-half were 0.966 and 0.941, respectively. Under the DCMs framework, the classification consistency reliability of nine attributes ranged from 0.81 to 0.99 with the average of 0.956. These results indicate that the DCT-IA has very good reliability based on both CTT and DCMs. It also shows good content validity given that it measures all IA symptom criteria defined in the DSM-5. In terms of the convergent validity, the test score of DCT-IA has a correlation of 0.870 (*p* < 0.001) with the test score of IAT (Young, 1998b) and a correlation of 0.924 (*p* < 0.001) with the test score of APIUS (Lei and Yang, 2007). The PPIA based on DCMs has a correlation of 0.817 (*p* < 0.001) with test score of IAT (Young, 1998b), and a correlation of 0.767 (*p* < 0.001) with test score of APIUS (Lei and Yang, 2007).

**Figure 1:**
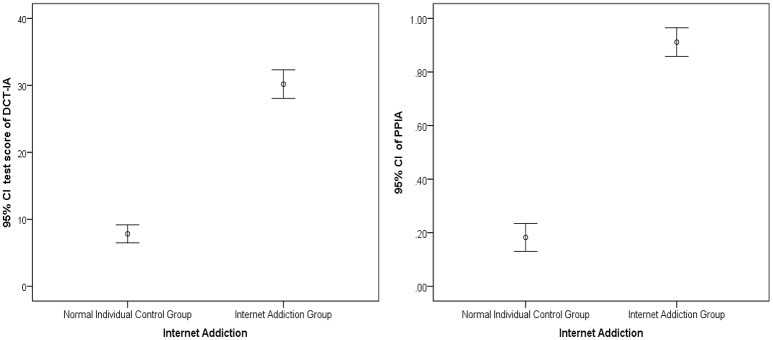
Error Bar Graph of the DCT-IA scores and PPIA for validation sample. 95% CI, 95% confidence interval. PPIA, posterior probability of internet addiction, which was calculated based on the DCT-IA and the diagnostic criteria in DSM-5 via DCMs.

To further examine its validity, the DCT-IA was administered to a validation sample consisting of healthy control group (*N*_1_ = 199) and IA group (*N*_2_ = 96) for cross-validation. Figure 1 shows the error bar of the DCT-IA scores and the PPIA, which is the probability of processing five or more symptom criteria of IA defined in DSM-5, for the two groups. There were clear different DCT-IA scores and PPIA between the two groups and the distributions were also reasonably symmetric within two groups. More specially, the IA group has a mean DCT-IA score of 30.19 (*SD* = 10.54), while the healthy control group has a mean 7.82 (*SD* = 9.56). A statistically significant difference in group means [*t*_293_ = 18.25, *p* < 0.001] was found with an effect size (Cohen's *d*) of 2.23. There was also a significant difference in the mean PPIA for the IA group (Mean = 0.912, *SD* = 0.263) and the healthy control group (Mean = 0.182, *SD* = 0.374); *t*_293_ = 17.13, *p* < 0.001, Cohen's *d* = 2.26.

Based on the validation sample, the sensitivity and specificity for predicting IA were 0.935 and 0.815 respectively using the 50% PPIA based on the DCT-IA and DSM-5 via DCMs, that is to say those who have over 0.5 PPIA is defined as IA. The diagnostic odds ratio is 45.30 (*P* < 0.001) with a 95% confidence interval of [20.82, 98.54]. Additionally, the area under ROC curve (i.e., AUC = 0.919) is high, indicating that the DCT-IA has considerable power to distinguish IA individuals and healthy individuals.

### Diagnostic score reporting

**Figure 2:**
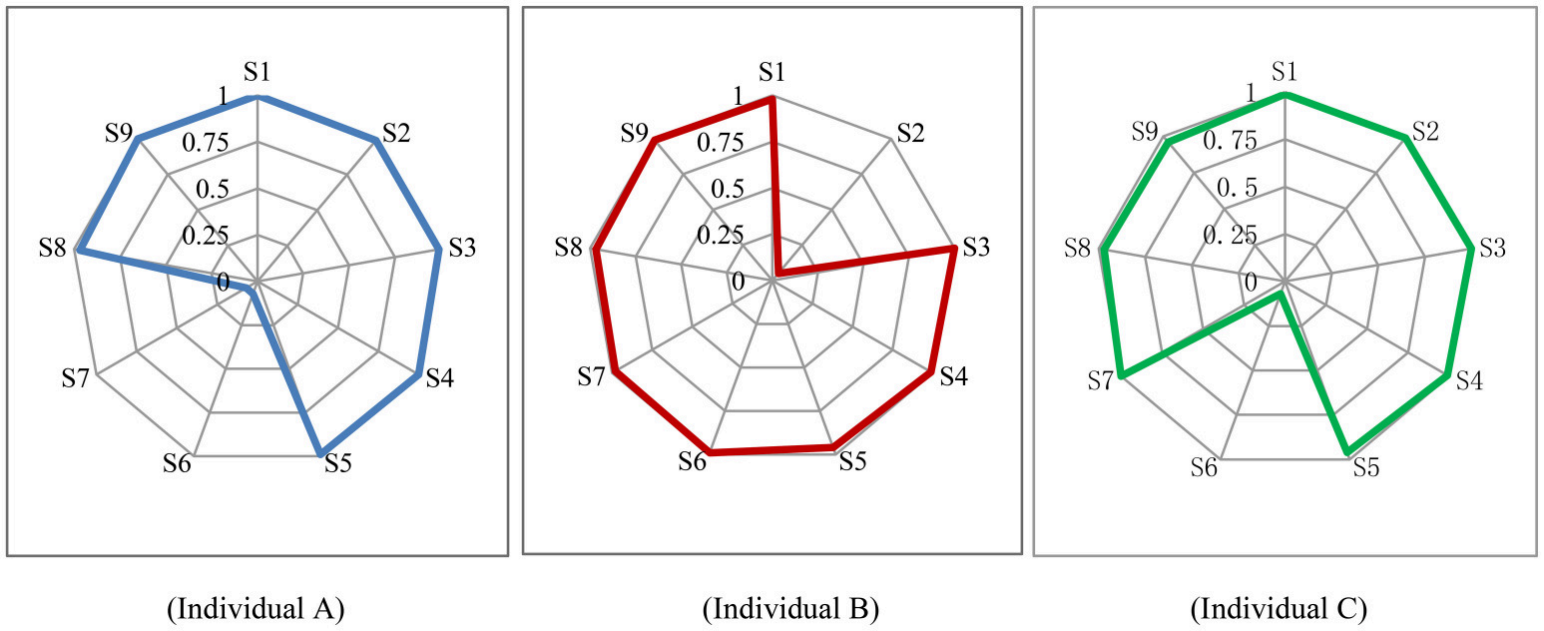
Symptom Spectrum of internet addiction for three individual. S1–S9 represent nine symptom criteria for internet addiction in DSM-5 in Table 1.

To demonstrate the unique information provided by DCMs, detailed score reports for three individuals were provided as an example in Table 5 and Figure 2. They were chosen in that they got the same test score in the IAT (Young, 1998b) and all defined as IA by IAT (Young, 1998b). Figure 2 shows the posterior probability that each symptom criterion has been satisfied for these individuals. Based on these probabilities, the PPIA for each individual can be calculated (see Table 5).

**Table 5:** Individual example estimates.

**Symptom criterion**	**Individuals**		
	**A**	**B**	**C**
S1	1.00	0.98	0.99
S2	0.99	0.05	0.99
S3	0.99	1.00	1.00
S4	1.00	0.99	0.99
S5	0.99	0.96	0.96
S6	0.07	0.99	0.07
S7	0.07	0.98	1.00
S8	0.97	0.97	0.97
S9	1.00	0.99	0.96
PPIA	1.00	0.97	1.00

*S1–S9 represent nine symptom criteria for internet addiction defined in DSM-5 in Table 1; PPIA, posterior probability of internet addiction, which was calculated based on the DCT-IA and the diagnostic criteria in DSM-5 via DCMs*.

Individual A, B, and C are all classified as IA by the DCT-IA (with the PPIA of 1.00, 0.97, 1.00, respectively), which is consistent to the diagnosis of IAT (Young, 1998b). However, they differ in their symptom profiles. From Figure 2 and Table 5, Individual A (male, 17 years old and from county) probably satisfies seven symptoms except for S6 and S7; while Individual B (male, 14 years old and from city) probably satisfies eight symptoms except for S2; and Individual C (female, 13 years old and from county) probably satisfies eight symptoms except for S6. These detailed report may be very valuable for diagnostic and interventional purposes.

## Discussion

In this study, a new instrument tool for IA, the DCT-IA, is developed using DCMs based on DSM-5. Results of this study reveal that, the DCT-IA has good reliability and validity, and high sensitivity and specificity. For example, the DCT-IA measures all nine symptom criteria of IA in DMS-5. It also shows the power to distinguish IA individuals and healthy individuals in the validation sample. Item psychometric properties were examined as well. For instance, to obtain accurate individuals' score reports, the most appropriate DCMs were selected empirically and used for different items, and some items were excluded due to low item discrimination, differential item functioning between different groups or poor item-fit.

Another contribute of this study is that the cutting-edge psychometric theory (i.e., DCMs) were firstly employed for assessment of internet addition to obtain more accurate, valid, and rich information. DCMs are promising in guiding test development and detailed score reporting in psychological assessment. Unlike CTT and IRT models, DCMs typically take the complicated interactions between multiple fine-grained latent variables into account, which allows considerable flexibility in modeling respondents' item responses. As shown in this study, DCMs can be used not only to evaluate psychometric properties for test and items but also to provide diagnostic information at both the generic diagnostic information and the symptom level diagnostic information.

There were also some limitations about this study. Firstly, the used method was more complicated than CTT and IRT. For example, the G-DINA model was very complex and had lots of parameters to be estimated. Therefore, user-friendly software should be developed in future (de la Torre et al., 2015). Secondly, all items were modified to two category responses (yes or on) even though some items were interval Likert-type responses in their raw inventories, which might cause a loss of information. Thirdly, the proposed method in this study was only used to analyze the variables with two-category response scale. However, it can be easily extended to interval Likert-type scale given that the polytomously-scored G-DINA model had been developed by Ma et al. (2016).

Despite promising results, to unlock the potential of the DCMs, more researches are needed. Although high sensitivity and specificity were observed in current study, it is still necessary to further validate the findings using large samples to help stabilize the estimation of the sensitivity and specificity. Another limitation is that, as noted by Gibbons et al. (2012), to decrease patients' burden, a short test that can be administered quickly is important. They showed that the number of items administered can be significantly reduced without the loss of estimation accuracy through computerized adaptive testing (CAT). Therefore, further studies may explore how to combine DCMs and CAT to obtain accurate results via a short test.

## Conclusion

This study developed a DCT-IA based on DCMs, a cutting-edge psychometric theory, to obtain accurate, valid, and rich information from the questionnaires or instruments for IA. Different from traditional questionnaires, the newly developed questionnaire can simultaneously provide general-level diagnostic information about the PPIA, and the detailed symptom criteria-level information about the probability of having each symptom criterion defined in DSM-5 for each person. This information gives insight into tailoring individual-specific treatments for IA, and could potentially increase these treatments' effectiveness.

## Ethics statement

This study was carried out in accordance with the recommendations of ethics committee of Center for Mental Health Education and Research of Jiangxi Normal University with written informed consent from all subjects. All subjects gave written informed consent in accordance with the Declaration of Helsinki. The protocol was approved by the ethics committee of Center for Mental Health Education and Research of Jiangxi Normal University.

## Author contributions

DT: design of the study, data collection, data analysis, paper writing, and revision. XG and DW: data analysis and interpretation of data for the work. YC: data analysis, interpretation of data for the work, and paper revision.

### Conflict of interest statement

The authors declare that the research was conducted in the absence of any commercial or financial relationships that could be construed as a potential conflict of interest.
